# Pirfenidone Attenuates Fibrosis and Neovascularization in 3D Spheroid‐Laden Hydrogel Culture

**DOI:** 10.1155/term/5557686

**Published:** 2026-04-15

**Authors:** Rayan Abdulhadi, Jorge Rodrigo Pintado, Mohammed AbuAlia, Shadi Motamed, Meghan Moran, Marcella K. Vaicik, Markus A. Wimmer, Anna Plaas, Georgia Papavasiliou

**Affiliations:** ^1^ Department of Biomedical Engineering, Armour College of Engineering, Illinois Institute of Technology, Chicago, Illinois, USA, iit.edu; ^2^ Department of Chemical and Biological Engineering, Armour College of Engineering, Illinois Institute of Technology, Chicago, Illinois, USA, iit.edu; ^3^ Department of Orthopedic Surgery, Rush University Medical Center, Chicago, Illinois, USA, rush.edu; ^4^ Department of Anatomy and Cell Biology, Rush University Medical Center, Chicago, Illinois, USA, rush.edu; ^5^ Department of Internal Medicine, Division of Rheumatology, Rush University Medical Center, Chicago, Illinois, USA, rush.edu

**Keywords:** arthritides, fibrosis, hydrogels, neovascularization, spheroid culture

## Abstract

Fibrosis and angiogenesis are key contributors to synovial inflammation in both the early and progressive stages of rheumatoid arthritis (RA) and osteoarthritis (OA), making them important therapeutic targets to mitigate joint tissue damage. In vitro drug screening, particularly for antifibrotic and antiangiogenic efficacy, is a standard method for evaluating therapeutic candidates prior to in vivo testing. Traditionally, most studies have relied on two‐dimensional (2D) monolayer cell cultures, which lack physiologically relevant cell–matrix and cell–cell interactions. Substantial evidence now indicates that three‐dimensional (3D) culture systems more accurately recapitulate the structural and functional complexity of native tissue environments. We employed 3D spheroid culture models of fibrosis and neovascularization to evaluate the antiangiogenic and antifibrotic effects of pirfenidone (PFD), an FDA‐approved drug for idiopathic pulmonary fibrosis. Spheroid monocultures of 3T3 fibroblasts and co‐cultures of human umbilical vein endothelial cells (HUVECs) and human aortic smooth muscle cells (SMCs) were encapsulated in cell‐adhesive, proteolytically degradable polyethylene glycol (PEG) hydrogel scaffolds. The temporal effects of PFD dose and timing of addition in culture on fibroblast outgrowth, vascular sprouting, and viability were quantified up to 14 days. PFD treatment led to dose‐dependent inhibition of both fibroblast outgrowth and vascular sprouting, depending on the initial timing of PFD addition, with cell viability maintained under all conditions. In addition, PFD reversed the onset of fibrosis and neovascularization. PFD exhibited antifibrotic activity and antiangiogenic potential in 3D cultures.

## 1. Introduction

In vitro cell culture assays are standard methodologies to assess drug toxicity and efficacy prior to in vivo preclinical or clinical testing. Typically, such assays have been performed using two‐dimensional (2D) cell cultures, either on tissue culture‐treated polystyrene or other planar surfaces. Recently, it has been recognized that such studies involving 2D cell culture have been inconsistent with in vivo findings of drug specificity and efficiency. This is primarily attributed to the absence of cell–cell and cell–extracellular matrix (ECM) interactions in 2D culture that are unique functions of differentiated cell types and hence impact drug responsiveness within the in vivo tissue microenvironment [1–3]. As a result, three‐dimensional (3D) cell culture platforms, where cells are embedded within modified hydrogel matrices, are now finding widespread applications in drug discovery [3, 4]. Spheroid‐laden hydrogel culture models, in particular, allow for intercellular communication, cell–ECM interactions, and the spatial organization of cells [5]. Thus, over the past two decades, 3D spheroids comprised of cell monocultures or co‐cultures have been widely utilized as in vitro models for drug development for treatments of cancer [6], bone mineralization [5], fibrosis [7], angiogenesis [8–13], and synovial hyperplasia [14].

Pirfenidone (PFD) is an antifibrotic drug, with anti‐inflammatory activities [15] used in clinical practice for treatment of idiopathic pulmonary fibrosis, but has been shown to also halt fibrotic progression in other organs including the liver [16], heart [17], and kidney [18, 19]. The antifibrotic action is not only primarily targeted to disrupt TGFβ1 signaling and downstream collagen production [20, 21] but also includes modulation of the JAK2/STAT3 pathway [22] to prevent angiogenesis [23]. Our previous in vivo findings demonstrated that oral administration of PFD alleviates synovial fibrosis in animal models of post‐traumatic osteoarthritis (OA) and slowed OA disease progression [24]. Studies by others have demonstrated that oral gavage administration of PFD attenuates synovial fibrosis and inflammation and OA progression in vivo [25] as well as synovial pannus formation by reducing inflammatory cell infiltration and angiogenesis [22]. Together these studies underscore the importance of repurposing PFD to slow down or prevent OA and RA disease progression.

With this perspective in mind, we utilized spheroid‐laden hydrogels to investigate the in vitro effects of the clinically available antifibrotic drug, pirfenidone (5‐methyl‐1‐phenyl‐2‐(1H)‐pyridine, PFD), in attenuating fibrosis and neovascularization, processes recognized to be integral to the pathology of degenerative joint diseases including rheumatoid arthritis (RA) and OA. In both conditions, the synovium plays a central role in disease progression. In RA, infiltrates of inflammatory cells into the synovial tissues stimulate fibroblast proliferation, transformation, and neovascularization, converging in the formation of an aggressive pannus [26–29]. Clinical and animal model studies of OA [30, 31] have shown that synovial fibroblasts, adipose resident stromal cells, and innate macrophages are activated following intra‐articular soft tissue injury [32], resulting in neovascularization and associated fibrotic remodeling within the synovium and soft tissues of affected joints [33–52]. Given that existing treatments for both OA and RA primarily are directed to control pain and inflammation [53, 54] as well as the critical role of the synovium in driving fibrosis and angiogenesis in both diseases, targeted delivery of PFD to the synovium would be a desirable methodology to mitigate these pathological processes to slow and/or prevent further joint damage.

Our motivation for this study was to employ a 3D spheroid polyethylene glycol (PEG) hydrogel culture platform that supports a robust proangiogenic and profibrotic microenvironment, two hallmark processes of synovial pathogenesis in RA and OA that cannot be reproduced in 2D culture. We then sought to evaluate how PFD dosing and timing influence the mitigation of these pathological cell behaviors. The PEG hydrogel matrix is functionalized with RGD and MMP‐sensitive peptide sequences at concentrations previously shown by us to support fibroblast invasion [55] and neovascularization [10] of encapsulated spheroids over a range of Young’s modulus values of soft tissues, including that reported for rat, porcine, and human synovium [56]. It must be noted that the presented 3D spheroid culture platform is not intended to recapitulate the complete biochemical composition or full spectrum of mechanical properties of healthy and diseased synovium, the values for which vary widely in literature depending on anatomical location in the joint and measurement methods used [56–58]. Instead, our presented spheroid culture platform aims to identify the effects of PFD dose and timing of its administration on fibroblast invasion and vascular sprouting within a 3D hydrogel matrix to optimize concentration and time frames over which the drug mitigates these synovium‐relevant cellular behaviors prior to in vivo studies.

To date, in vitro mechanistic studies of the antifibrotic and antiangiogenic effects of PFD have been primarily limited to 2D culture of various cell types including human fibroblast‐like synoviocytes (FLS) [25], human fetal lung fibroblasts [21], human Tenon’s fibroblasts [59], human lung microvascular endothelial cells [60], human pulmonary artery smooth muscle cells (SMCs) [60], human umbilical vein endothelial cells (HUVECs) [22, 59], and hybrid cell lines combining HUVECs and A549 lung carcinoma cells (EA.hy926) [61]. In 3D multicellular culture models of cardiac fibrosis, comprised of individual cell suspensions of stem cell‐derived cardiomyocytes and human fetal cardiac fibroblasts embedded in a gelatin methacryloyl hydrogel, PFD was found to significantly reduce the expression of key fibrotic markers [62]. To our knowledge, however, the antifibrotic and antiangiogenic responsiveness to PFD treatment in 3D spheroid culture have not been previously explored.

In this study, the effects of PFD in attenuating fibroblast outgrowth and vascular sprouting in 3D culture were evaluated in spheroid‐laden PEG hydrogel scaffolds immobilized with the integrin‐binding RGD peptide ligand and proteolytically degradable MMP‐sensitive peptide domains. Fibroblast (3T3) spheroids or (HUEVC)/human aortic SMC spheroid co‐cultures were encapsulated within the cell‐adhesive, proteolytically degradable PEG hydrogels using visible light free‐radical photopolymerization [9–13, 55]. This chemistry allows for rapid and cytocompatible encapsulation of spheroids within the crosslinked hydrogel network with highly reproducible matrix properties. We have previously identified the optimal combination of matrix cues including modulus, proteolytic degradation rate, and immobilized RGD concentration that maximize rates of neovascularization within 3D spheroid culture using PEG scaffolds [9, 10]. In the present study, spheroid‐laden PEG hydrogels were engineered with these matrix cues to evaluate the in vitro efficacy of (1) PFD dosage and (2) timing of administration in mitigating fibrosis and vascular sprouting over a period of 14 days (D14). The experimental design of our in vitro approach, devised to establish foundational insight of the therapeutic potential of PFD in attenuating fibrosis and neovascularization associated with inflamed synovial tissues is depicted in Figure 1(a). The corresponding dosing regimen for both immediate and delayed PFD administration groups over the 14‐day experimental timeline is illustrated in the schematic in Figure 1(b).

FIGURE 1Overview of experimental design for evaluating PFD dose and timing of administration on fibrosis and neovascularization in 3D spheroid culture. (a) 3T3 fibroblast monocultures and SMC/HUVEC coculture spheroids are formed using methyl cellulose using a total of 20 K cells/spheroid. Each spheroid type is encapsulated in a cell adhesive (RGD) proteolytically degradable PEG hydrogel scaffold following visible‐light free‐radical photopolymerization. (b) PFD is supplemented in culture media at different concentrations starting at Day (D) 1 or D4 following spheroid encapsulation and replenished every other day until D14.

**Figure:**
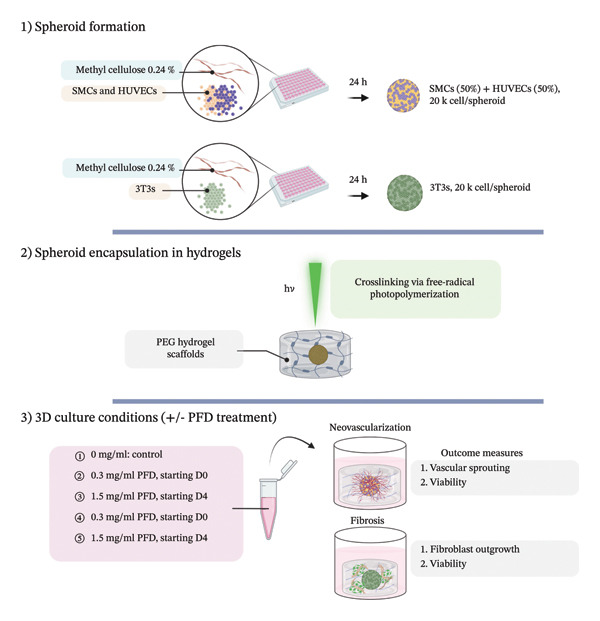
(a)

**Figure:**
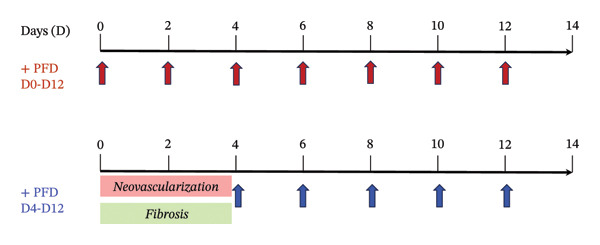
(b)

## 2. Materials and Methods

### 2.1. Materials

Reagents for peptide synthesis, such as Fmoc‐amino acids and Wang resins were obtained from AAPPTec. Dimethylformamide (DMF), diethyl ether, O‐benzotriazole‐N,N,N′,N′‐tetramethyluronium hexafluorophosphate (HBTU), piperidine, phenol, trifluoroacetic acid (TFA), 1‐methyl‐2‐pyrrolidinone (NMP), N,N‐diisopropylethylamine (DIEA), thioanisole, and triisopropylsilane (TIS) and HPLC‐grade acetonitrile were purchased from Sigma‐Aldrich. Methylcellulose was purchased from Sigma‐Aldrich. PFD (purity > 98%) was purchased from TCI America live/dead assays and confirmed by UV/VIS absorbance. Calcein AM and ethidium homodimer‐1, used for live/dead cell staining of spheroids, were purchased from Invitrogen. Hydrogel precursor components, including N‐vinyl‐2‐pyrrolidone (NVP), triethanolamine (TEA), and eosin Y were obtained from Sigma‐Aldrich, and acrylate‐poly(ethylene) glycol‐succinimidyl valerate (acryl‐PEG‐SVA, 3.4 kDa and 5.0 kDa), were purchased from Laysan Bio.

### 2.2. Solid‐Phase Peptide Synthesis (SPPS) of Cell Adhesive and MMP‐Sensitive Peptides

The cell‐adhesive integrin binding peptide (YRGDS) ligand and the MMP‐sensitive peptide featuring a repeat of the VPMSMR amino acid sequence, GGVPMS↓MRGDGVPMS↓MRGGK (DSite, 2007.4 Da), were synthesized using solid‐phase automated peptide synthesis (SPPS). SPPS was performed on a Focus Xi peptide synthesizer (AAPPTec, Louisville, KY), utilizing Fmoc (9‐fluorenylmethyloxycarbonyl) chemistry. Peptide synthesis protocols were optimized according to manufacturer (AAPPTec) guidelines. Briefly, peptides were elongated by coupling amino acids at 0.4 M concentration in NMP to a Wang resin in the presence of DIEA as the base and HBTU, the latter used for activation. Fmoc protecting groups were removed using 20% piperidine in DMF. Peptides were then cleaved from the resin with a cocktail consisting of TFA:TIS:DIW:thioanisole:phenol at a ratio of 90:2.5:2.5:2.5:2.5 for 2.5 h at the highest rocking speed at 4°C. Crude peptides were then precipitated in ice‐cold diethyl ether and dried overnight and purified using an Agilent 1200 series HPLC system with a C18 Waters column (19 × 100 mm). Peptide purity > 95% was confirmed by ion trap time‐of‐flight (IT‐TOF) mass spectroscopy. Purified peptides were lyophilized and stored at −20°C until use.

### 2.3. Synthesis of Photopolymerizable Cell Adhesive Peptide Macromers and Protease‐Sensitive Peptide Crosslinkers

Two distinct photopolymerizable acrylate macromers were synthesized to create cell‐adhesive and proteolytically degradable PEG hydrogel scaffolds. The synthesized macromers included a PEG diacrylate crosslinker that contained the DSite MMP‐sensitive peptide between the terminal acrylate groups of PEGDA (DSite‐PEGDA) and an RGD‐containing PEG monoacrylate macromer (RGD‐PEGMA). The peptide functionalized crosslinker (DSite‐PEGDA) and macromer (RGD‐PEGMA) were synthesized by conjugation of the DSite MMP‐sensitive peptide and the YRGDS peptide to acryl‐PEG‐SVA macromers of different molecular weight and molar ratio of peptide to acrylate derivatives. The DSite‐PEGDA crosslinker, Acryl‐PEG_5000_‐GGVPMS↓MRGDGVP‐PEG_5000_‐Acryl (MW ∼12 kDa), was formed by reacting the DSite peptide with acryl‐PEG‐SVA (5 kDa) at a 1:2 molar ratio allowing the peptide to be conjugated between the terminal acrylate groups to synthesize to enable its incorporation within network crosslinks following hydrogel formation. The RGD‐PEG‐MA macromer, Acryl‐PEG_3400_‐YRGDS (MW ∼4 kDa), was synthesized by conjugating the YRGDS peptide with acryl‐PEG‐SVA (3.4 kDa) at a 1.05:1 molar ratio to produce a monofunctional acrylate macromer to enable tethering of the peptide to the hydrogel network following crosslinking. Conjugation reactions were carried out in 50 mM sodium bicarbonate (NaHCO_3_) buffer at pH 8.0 with peptide added dropwise to acryl‐PEG‐SVA solutions, reacted for 4 h in the dark at 4°C and subjected to dialysis for 24 h to remove unreacted components using dialysis cartridges (MW cutoff = 7 kDa and 5 kDa for Acryl‐PEG_5000_‐GGVPMS↓MRGDGVP‐PEG_5000_‐Acryl and Acryl‐PEG‐YRGDS, respectively). Macromer acrylation efficiency was confirmed to be > 92 with^1^H NMR.

### 2.4. Quantification of Hydrogel Mechanical Properties

The storage modulus (*G*′) of PEG scaffolds was quantified using small amplitude oscillatory shear on a HR‐10 Discovery Hybrid rheometer (TA Instruments, Newcastle, DE). Following complete crosslinking, spheroid‐free PEG hydrogels were incubated in PBS for 48 h until equilibrium swelling was achieved. Hydrogels were then subjected to a constant strain amplitude of 0.05% at a frequency, *ω* = 10 rad/s, as these conditions yield rheological properties that fall within the linear viscoelastic regime where the hydrogel response is strain‐independent [63] reflective of intrinsic small‐deformation material properties of the PEG network [64]. A subsequent time sweep confirmed that G′ remained constant with time, indicating a stable viscoelastic response under the selected testing conditions and is included in Supporting Information (Supporting Figure 1).

The Young’s modulus (*E*) of the hydrogels was estimated from the average value of *G′* based on the isotropic and homogeneous material properties using the following equation:

(1) E=2G′1+ν,

where *ν* is Poisson’s ratio and equivalent to 0.5 for crosslinked PEG hydrogels assuming negligible volume changes during deformation [65].

### 2.5. Quantification of Hydrogel Degradation Kinetics

The degradation profiles of protease‐sensitive hydrogels were characterized using gravimetric measurements. Fully swollen hydrogels were incubated in an interstitial collagenase IA solution (*Clostridium histolyticum*) (125 CDU/mg) at a concentration of 0.1 μg/mL in 10 mM HBS containing 1 mM CaCl_2_ (pH 7.4) at 37°C. Weights of fully swollen hydrogels were recorded at predetermined time intervals every 30 min, up to 7 h. At each time point, the collagenase solution was removed and replaced with fresh enzyme solution to maintain enzymatic activity. This process was repeated until complete hydrogel degradation was achieved.

### 2.6. Characterization of Hydrogel Network Mesh Dimensions

The mesh dimensions of PEG hydrogel scaffolds are critical to the size and conformation of molecules and their diffusivity through the network. These dimensions were calculated from gravimetric measurements in hydrogel swelling using two methodologies. The first methodology involved the use of the Flory–Rehner equation [66–68] to predict the mesh size (*ξ*) using the Canal and Peppas equation [69]. The methodology was based on an approach we recently developed that builds off of the Flory–Rehner equation but which specifically accounts for the molecular weight of the PEG crosslinker and its deformation under swelling [70], neglected by Flory–Rehner [67, 68, 71]. This results in estimation of two mesh dimensions including the intrachain mesh dimension (*ξ*_*I* *C*_) defined as the average distance between two crosslink points on the same primary chain that have one crosslink point between them, and the interchain mesh dimension (*ξ*_*N* *C*_) defined as the average distance between two crosslink points on different primary chains [70]. For both methodologies, gravimetric measurements of fully swollen hydrogels (*m*_*S*_) 24 h post‐photopolymerization and the mass of dried hydrogels (*m*_*D*_) following lyophilization for 48 h were used to estimate mesh sizes from the mass swelling ratio (*Q*_*m*_):

(2) Qm=mSmD.

The value of the average molecular weight between crosslinks, ${\overset{¯}{M}}_{c}$, was estimated based on the classical Flory–Rehner theory according to the following equation:

(3) 1Mc¯=ν¯/V1¯ln1−ϕ2,s+ϕ2,s+χ1ϕ2,s2ϕ2,s−13/−ϕ2,s/2,

where $\overset{¯}{\nu }$ represents the specific volume of the polymer (0.893 cm^3^/g), $\overset{¯}{V_{1}}$ is the specific volume of the solvent (18 cm^3^/g), *χ*_1_ (0.426) is the polymer–solvent interaction parameter, and *ϕ*_2_,_*s*_ is the polymer volume fraction of the hydrogel in the swollen state. The latter is equivalent to the reciprocal of the volumetric swelling ratio (*Q*_*V*_) which can be calculated from *Q*_*m*_, the density of the solvent (*ρ*_1_ = 0.997 g/cm^3^ for H_2_O), and the density of the polymer (*ρ*_2_ = 1.3 g/cm^3^) as follows:

(4) ϕ2,S=1QV=1/ρ2Qm/ρ1+1/ρ2 .

Values of ${\overset{¯}{M}}_{c}$ provided an estimation of the classical mesh dimension (*ξ*_*F* *R*_) of the swollen hydrogel network, calculated using the Canal and Peppas equation [69]:

(5) ξFR=ϕ2,s−13/ l Cn12/2Mc¯Mr12/,

where *l* represents the average bond length between the C‐C and C‐O bonds in the PEG repeat unit (*l* = 1.46 Ǻ), *C*_*n*_ is the characteristic ratio of PEG (*C*_*n*_ = 4), and *M*_*r*_ is the molecular weight of the PEG repeat unit (*M*_*r*_ = 44 g/mol). Values for *ξ*_*I* *C*_ and *ξ*_*N* *C*_ were calculated from swelling ratio measurements and estimations of the crosslinker fraction in the polymer using equations described in our previously published approach [70, 72]. Conservative estimates of the diffusion coefficient of PFD in the swollen hydrogel (*D*_*g*_) were obtained from the Lustig–Peppas expression [73]:

(6) DgD0=1−rsξFRexp  −Yϕ2,s1−ϕ2,s,

where *r*_*s*_ and *Y* represent the Stokes (hydrodynamic) radius of PFD (0.465 nm) and the ratio of the critical volume required for translational movement of the encapsulated drug molecule and the average free volume per molecule of solvent, respectively. A reasonable approximation for *Y* is unity. The Stokes–Einstein equation was used to estimate the diffusion coefficient of PFD in the pure solvent (water), *D*_0_, using Boltzmann’s constant (*k*_*B*_ = 1.38 × 10^−23^ J/K), the absolute temperature (*T* = 310.15 K), and the dynamic viscosity of water (*η* = 0.89 MPa·s) as follows:

(7) D0=kBT6 πηrs.

### 2.7. Kinetic Measurements of PFD Scaffold Uptake

The diffusive uptake of PFD in PEG scaffolds was quantified using over D14 using UV/VIS absorbance (Supporting Figure 2). PFD was dissolved in 50 μL of ethanol and then added to the media. After hydrogels were formed in 96‐well plates, a volume of 0.5 mL of PFD solution (1 mg/mL) was added to each well and the medium was removed and replenished with fresh PFD solution every other day up to D14. This experiment emulated PFD media supplementation and scaffold uptake in the 3D spheroid culture experiments. At pre‐determined times (0, 2, 4, 7, and 14 days), hydrogels were subjected to degradation following exposure to 60°C and pH 10 with the addition of 0.1 mM NaOH. The degraded solution was adjusted to pH 7.5 using HCl and the concentration of PFD uptake in the scaffold was quantified from absorbance readings of the degraded solution. Absorbance readings were subtracted from solutions of degraded hydrogel incubated in PBS for D14 to correct for background signal.

### 2.8. Preparation of Suspension Culture Media for Spheroid Formation

Methyl cellulose stock solution was prepared as described previously [74]. Briefly, 1.2 g of methylcellulose powder was autoclaved in a flask under stirring and dissolved in 50 mL either endothelial cell growth medium‐2 (EGM‐2, Lonza) or DMEM supplemented with 10% FBS and 1% penicillin‐streptomycin (Fisher Scientific), depending on desired spheroid type (see below) at 60°C for 20 min under continuous stirring. Subsequently, an equal volume of pre‐warmed medium was added and stirred overnight at 4°C. Post‐mixing, the stock solution was cleared of cellulose fibers by centrifugation at 4000 rpm for 4 h at 4°C. The clear, viscous supernatant, constituting 90%–95% of the original solution, was transferred into two 50 mL sterile tubes to be utilized for spheroid formation.

### 2.9. Formation of Fibroblast Monoculture and Vascular Co‐Culture Spheroids

HUVECs (Lonza) were cultured using an endothelial cell growth medium‐2 (EGM‐2, Lonza) BulletKit and human aortic (SMCs (Lonza) and NIH/3T3 fibroblasts (ATCC) were cultured in Gibco 4 g/L glucose DMEM supplemented with 10% FBS and 1% penicillin‐streptomycin (Fisher Scientific). NIH 3T3 murine spheroid fibroblast monocultures (20,000 cells total/well, passages 2‐3) were created following suspension in DMEM media (150 mL) with 0.24% (w/v) methyl cellulose, transferred to round‐bottomed, noncell‐binding 96‐well plates and spheroid formation was then allowed to proceed for 48 h at 37°C with 5% CO_2_. Vascular co‐culture spheroids were established similar to fibroblast spheroid formation by suspending HUVEC (passages 2–5) and SMC (passages 2–5) at a 1:1 cell ratio (20,000 total cells/well) in EGM‐2 culture media (150 mL) with 0.24% (w/v) methyl cellulose.

### 2.10. Spheroid‐Laden Hydrogel Synthesis by Free‐Radical Photopolymerization

Fibroblast monoculture or HUVEC/HUASMC co‐culture spheroids were separately encapsulated into PEG hydrogels using visible light (*λ* = 514 nm) free‐radical photopolymerization as described by our prior study [10]. Hydrogel precursor solutions were prepared in 1 × phosphate‐buffered saline (PBS, pH 7.4), containing 37 mM NVP, 7 mM TEA co‐initiator, and 0.01 mM eosin Y photosensitizer, with 2 mM Acryl‐PEG_5000_‐Dsite‐PEG_5000_‐Acryl crosslinker and 5 mM Acryl‐PEG_3400_‐YRGDS. The pH of the precursor solution was adjusted to 7.4 following the addition of HCl. A hydrogel base layer was first formed by photopolymerizing a precursor volume of 50 μL in a flat‐bottom 96‐well plate for 5 min using an argon ion laser (Coherent Inc., Santa Clara, CA) at an intensity of 100 mW/cm^2^. A single pre‐formed spheroid was then placed on top of the hydrogel base layer. Subsequently, a precursor volume of 50 μL was added on top of the base layer to fully cover the spheroid and photopolymerized under similar conditions as described above [75].

### 2.11. Quantification of Temporal 3D Spheroid Fibroblast Outgrowth and Vascular Sprouting in Response to PFD Treatment Using Noninvasive Phase Contrast Imaging

To determine if the addition of PFD prevents and reverses fibrosis and/or neovascularization in 3D culture in a dose dependent manner, PFD was introduced at concentrations of 0.3 and 1.5 mg/mL in culture medium starting either immediately following spheroid encapsulation in scaffolds at D0 or at D4 after encapsulation when extensive fibrosis and vascular sprouting were established. For all cases investigated and throughout the duration of the 3D culture, fresh PFD (at either 0.3 or 1.5 mg/mL) was added with culture medium changes every 48 h up to D14. Temporal 3D vascular sprouting responses and fibroblast outgrowth were monitored and quantified from acquired 2D phase contrast microscopy images captured at D0, D7, and D14 following spheroid encapsulation as described in the subsequent section. The selected times facilitated assessment of early and progressive changes in spheroid responses to PFD treatment.

The extent of fibroblast outgrowth and vascular sprouting of spheroids in response to PFD dose and initial timing of its addition in 3D culture was quantified using a noninvasive imaging technique developed based on a previously reported segmentation method [76]. Traditional methods such as manual tracing are time‐consuming and inconsistent or rely on the use of fluorescent markers to quantify spheroid parameters in real‐time. Here, a sequence of image processing steps was used to enhance the visibility of structural edges of the spheroid images acquired via phase contrast microscopy (Carl Zeiss MicroImaging, Inc.), followed by an intensity‐based thresholding method. First, a Gaussian point spread function (PSF) was generated using an initial guess estimate to model the blurred phase contrast image. This estimate was then refined iteratively using the Richardson–Lucy algorithm. The updated PSF was used to perform deconvolution on the acquired images, which significantly reduced blurring of the structures outside the focal plane. Subsequently, a Sobel filter was used to further highlight these edges (Supporting Figure 3B) compared to the originally obtained image (Supporting Figure 3A). Intensity‐based thresholds were then established using the inflection points of the smoothed intensity curve derived from a row of pixels running horizontally through the middle of the image. This method effectively segmented the spheroid into its core and the area of outward growth (Supporting Figure 3C). For our analysis, we used indices of the area of the entire spheroid at D14, normalized to the spheroid area on D0.

### 2.12. Confocal Imaging for Quantification of 3D Spheroid Viability and Visualization of Fibroblast Outgrowth and Vascular Sprouting

To quantify 3D cell viability of fibroblast and vascular spheroids in response to PFD dosage and initial timing of administration, calcein AM and ethidium homodimer‐1 were used to stain live and dead cells, respectively, within spheroids at D14. At D14, culture media was removed from wells, hydrogels were rinsed with DPBS, and spheroid‐laden hydrogels were incubated with the stains at room temperature for 45 min according to manufacturer’s instructions. Live (green) and dead (red) cells were imaged using laser scanning confocal microscopy (Nikon A1 HD25, Nikon Inc.) and the percentage of live cells was quantified from the ratio of live to total pixels (representing live plus dead cells) from a series of z‐stack images that encompass the entirety of each spheroid. Calcein AM and ethidium homodimer‐1‐stained fibroblast and vascular spheroids were also visualized for 3D fibroblast outgrowth (Supporting Figure 4) and vascular sprouting (Supporting Figure 5) in response to PFD dose and initial timing of administration using confocal microscopy at D14.

### 2.13. Statistical Analysis

All experimental data are reported as the mean ± standard deviation. Two‐way analysis of variance (ANOVA) was employed to get statistical significance between experimental groups across one time point. Post hoc analysis was conducted using Tukey’s honest significant difference (HSD) test for multiple comparisons, utilizing GraphPad Prism software.

## 3. Results

### 3.1. Characterization of Hydrogel Scaffolds

**FIGURE 2:**
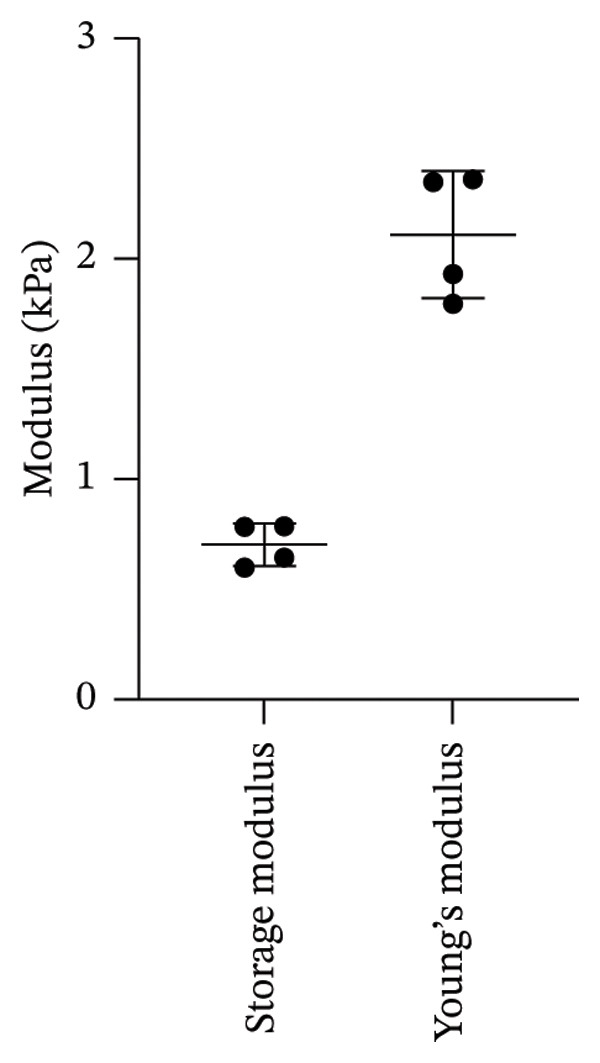
Mechanical characterization of PEG hydrogel scaffolds (*n* = 4).

**FIGURE 3:**
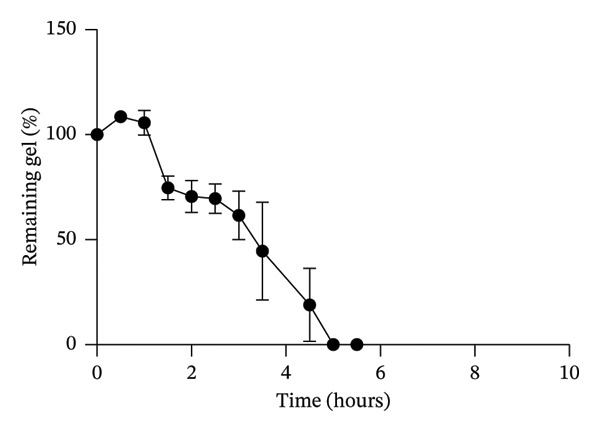
Degradation kinetics of protease‐sensitive PEGDA scaffolds based on gravimetric measurements in wet weight during collagenase incubation (*n* = 3).

In previous studies, we conducted a full factorial design of experiments (DOE) and identified the optimal combination of immobilized YRGDS cell adhesive peptide concentration, degradation rate, and modulus of PEG hydrogels that maximized vascular sprouting of HUVEC/SMC spheroids in 3D culture [10]. These scaffold conditions were utilized to promote robust in vitro fibrosis and vascular sprouting to investigate the impact of PFD dose and timing of addition in preventing and/or reversing these responses. Scaffolds were first characterized in terms of mechanical properties, in vitro degradation kinetics, and network mesh dimensions. Mechanical characterization of the hydrogels using rheometric measurements yielded a storage modulus of 0.703 ± 0.0986 kPa within the linear viscoelastic region under oscillatory shear deformation (Figure 2). The Young’s modulus was estimated as 2.11 ± 0.289 kPa from the acquired value of the storage modulus using a Poisson’s ratio of 0.5 (Figure 2). Incubation of scaffolds to activated collagenase solution revealed increases in hydrogel swelling in the initial 30 min of enzyme incubation followed by rapid decreases until due to degradation of crosslinks followed by complete material degradation at 7 h (Figure 3).

**FIGURE 4:**
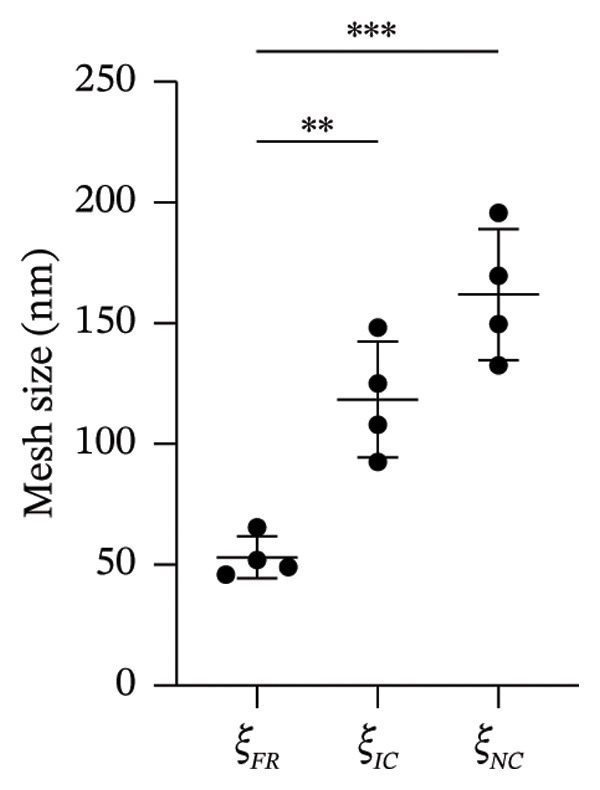
Comparisons of mesh dimensions of PEG hydrogels using the Flory–Rehner theory (*ξ*_*F* *R*_) and our modified Flory–Rehner approach accounting for the size of the crosslinking macromolecule (*ξ*_*I* *C*_ and *ξ*_*N* *C*_) (*n* = 4; ^∗∗^ *p* < 0.01, ^∗∗∗^ *p* < 0.001).

PEG hydrogels were characterized in terms of network mesh dimensions using the Flory–Rehner theory and new mesh dimensions we recently identified as relevant for hydrogels formed using crosslinking macromolecules [70, 77]. Using fully dry and swollen gel weights, the calculated average classical mesh size (*ξ*_*F* *R*_) quantified from swelling measurements using the classical Flory–Rehner theory and the Canal and Peppas equation [69] was equivalent to 81.3 ± 13.4 Å. The mesh dimensions calculated using our recently published approach [70] yielded intrachain (*ξ*_*I* *C*_) and interchain (*ξ*_*N* *C*_) mesh dimensions of 117.4 ± 19.6 Å and 146.3 ± 21.7 Å, respectively (Figure 4). Based on the quantified values of *ξ*_*I* *C*_ and *ξ*_*N* *C*_ and the estimated hydrodynamic diameter of PFD (9.26 Å) calculated using atomic Van der Waals volumes for spherical molecules in aqueous solution, the drug is capable of freely diffusing within the scaffold to interact with the cell spheroids to assess its effectiveness in mitigating fibrosis and neovascularization in 3D culture. These mesh dimensions are also larger than the reported hydrodynamic radii of essential growth factors in the media [78] which allows us to fully discern the effects of PFD in mitigating fibrosis and angiogenesis.

**FIGURE 5:**
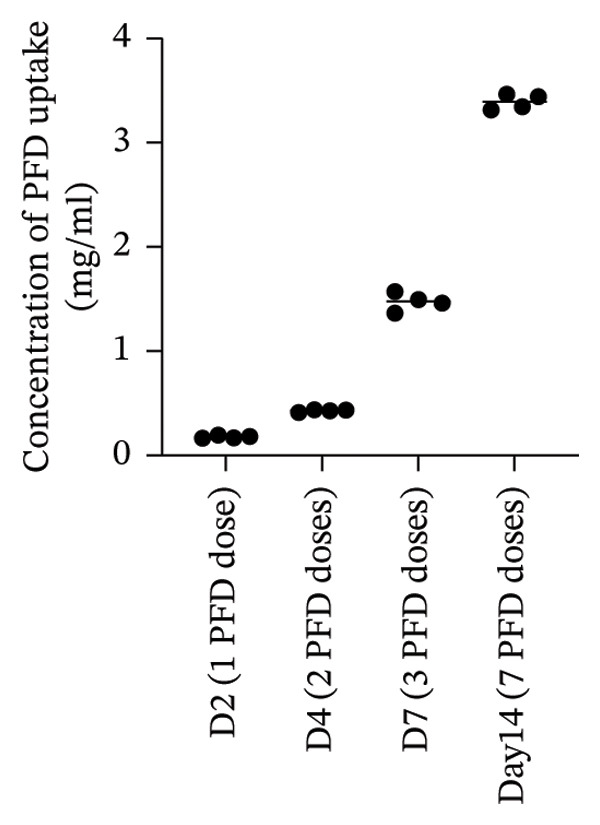
Kinetics of PFD diffusive scaffold uptake quantified following hydrolytic hydrogel degradation at 60°C and pH 10 and PFD absorbance measurements. PFD (1.0 mg/mL) was replenished every other day in the aqueous medium surrounding the scaffold from D0 until D14 (*n* = 4).

Measurements of PFD uptake kinetics into the hydrogels were performed over D14 by continuously replenishing the drug in the surrounding solution every other day, emulating the conditions used in 3D spheroid culture (Figure 5). PFD scaffold uptake exceeded cumulative concentration of 3 mg/mL, based on an initial PFD concentration of 1.0 mg/mL. Thus, the intrachain and interchain network dimensions enabled facile diffusion of the therapeutic into the scaffold.

### 3.2. PFD Attenuates Fibrosis and Neovascularization in 3D Culture

To evaluate the impact of PFD dosage on attenuation and/or reversal of fibroblast outgrowth and neovascularization in 3D spheroid cultures, PFD was added to the culture media at concentrations of 0.3 mg/mL or 1.5 mg/mL, doses previously identified to be therapeutically effective in 2D culture studies [59]. Media were supplemented with PFD every other day, beginning either at D0 or D4 post‐encapsulation up to D14. Our prior work has demonstrated that robust spheroid outgrowth and vascular sprouting responses are evident by D3. Accordingly, D0 and D4 were selected to determine whether PFD could attenuate early stage fibrotic and angiogenic responses once initiated.

In fibroblast spheroid cultures, the untreated control group exhibited a substantial increase in fibroblast outgrowth area from D0, reaching ∼77.3% by D14, consistent with robust cell proliferation (Figure 6). When PFD was administered starting at D0, fibroblast outgrowth was significantly reduced to ∼17.4% and ∼14.7% with 0.3 mg/mL and 1.5 mg/mL PFD, respectively, by D14 (Figure 6(b)). Similar findings of fibroblast outgrowth were observed at D7. This early PFD intervention did not exhibit a dose‐dependent effect. Conversely, when PFD was introduced at D4, a dose‐dependent reduction in outgrowth was observed: ∼40.1% and ∼16.2% for 0.3 mg/mL and 1.5 mg/mL, respectively, both significantly lower than the untreated control (Figure 6(c)) by D14. Similar dose‐dependent decreases were observed by D7. Qualitative visualization of attenuation in fibroblast outgrowth depending on PFD dose and initial timing of addition is further illustrated in the 2D flattened projections of confocal images shown in Figure 7. 3D confocal renderings acquired at D14 also indicate that a 1.5 mg/mL PFD dose attenuates fibroblast outgrowth even after being administered after D4 (Supporting Figure 4). For HUVEC/SMC spheroid co‐cultures, the control group (0 mg/mL PFD) exhibited an average increase in vascular sprouting area of 59.2% by D14 from D0 (Figures 8(b) and 8(c)). When PFD was added at D0, a dose‐dependent and significant reduction in vascular sprouting was observed by D7 and D14, with areas decreasing to ∼26.0% and ∼8.6% for 0.3 mg/mL and 1.5 mg/mL PFD, respectively (Figure 8(a)) by D14. Delayed addition of PFD starting at D4 also significantly reduced vascular sprouting to ∼28.9% and ∼21.4% relative to the untreated control for 0.3 mg/mL and 1.5 mg/mL PFD, respectively, by D14 (Figure 8(b)). In contrast to the dose‐dependent decrease in vascular sprouting response with PFD addition starting on D0, administration of the drug starting on D4 was not found to be dose‐dependent (Figure 8(b)). Qualitative visualization of attenuation in neovascularization based on PFD dose and initial timing of addition is further illustrated in the 2D flattened projections of confocal images shown in Figure 9. 3D confocal renderings acquired at D14 also indicate that a 1.5 mg/mL PFD dose attenuates vascular sprouting even after being administered after D4 (Supporting Figure 5). These results indicate that PFD attenuates both fibroblast invasion and neovascularization in 3D culture regardless of timing of administration and reverses fibrosis and vascular sprouting following its initial timing of addition at D4 at the higher PFD concentration of 1.5 mg/mL.

FIGURE 6(a) Representative phase contrast images of outgrowth of 3T3 fibroblast spheroids taken at D0, D4, D7, and D14 following encapsulation in scaffolds as a function of PFD dosing (0.3 and 1.5 mg/mL) and initial timing of PFD addition (D0 versus D4) in culture media (scale bar = 100 μm). Quantification of percent increase in fibroblast outgrowth area at D14 as a function of PFD dosage and initial timing of PFD addition at (b) D0 and (c) D4 (scale bar = 100 μm; *n* = 3; *p* < 0.0065 (^∗^), *p* < 0.0001 (^∗∗^)).

**Figure:**
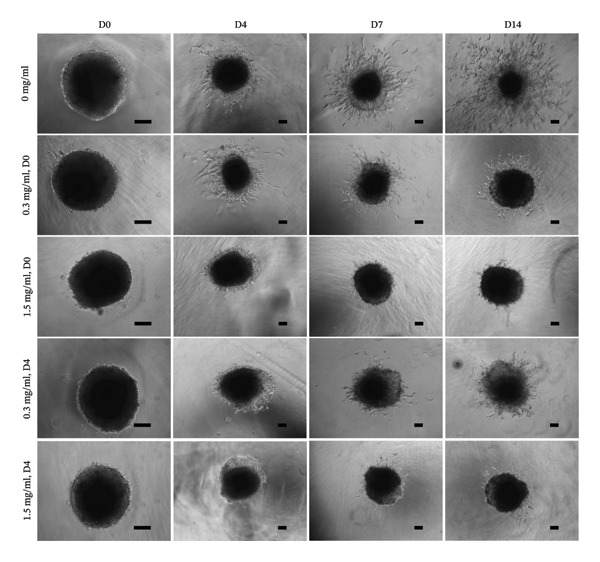
(a)

**Figure:**
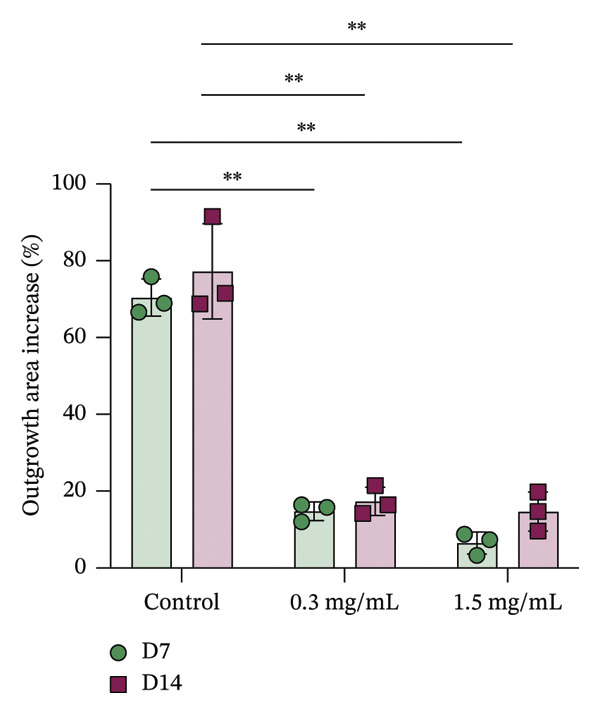
(b)

**Figure:**
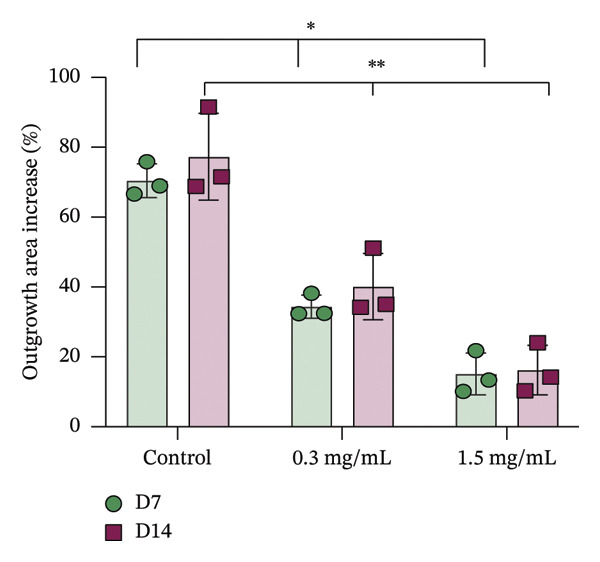
(c)

FIGURE 7Effect of PFD dose and timing of administration in culture media on fibroblast outgrowth and viability at D14. Two‐dimensional projections of z‐stack confocal images showing 3T3 fibroblast spheroid outgrowth under the following conditions: (a) 0 mg/mL PFD (control); PFD added on D0 at (b) 0.3 mg/mL and (c) 1.5 mg/mL; PFD added on D4 at (d) 0.3 mg/mL and (e) 1.5 mg/mL. (f) Quantification of the percentage of viable cells at D14 as a function of PFD dose and timing of administration. Viable and dead cells were visualized using calcein AM (green) and ethidium homodimer‐1 (EthD‐1, red), respectively (scale bar = 100 μm; *n* = 3; *p* < 0.05 (^∗^), *p* < 0.0001 (^∗∗^)).

**Figure:**
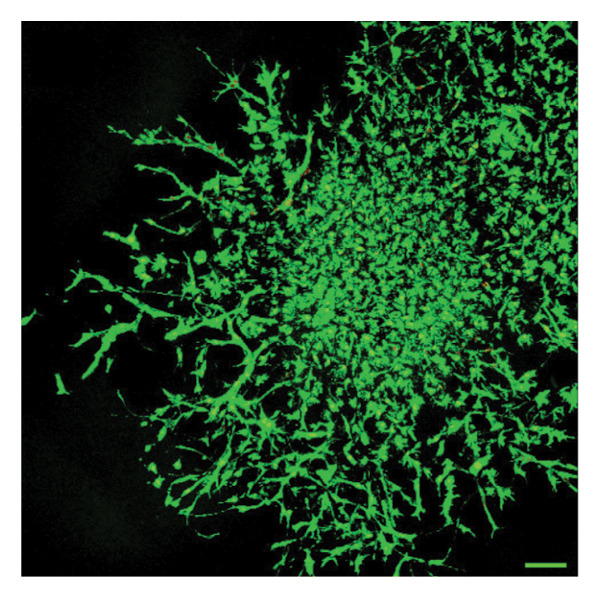
(a)

**Figure:**
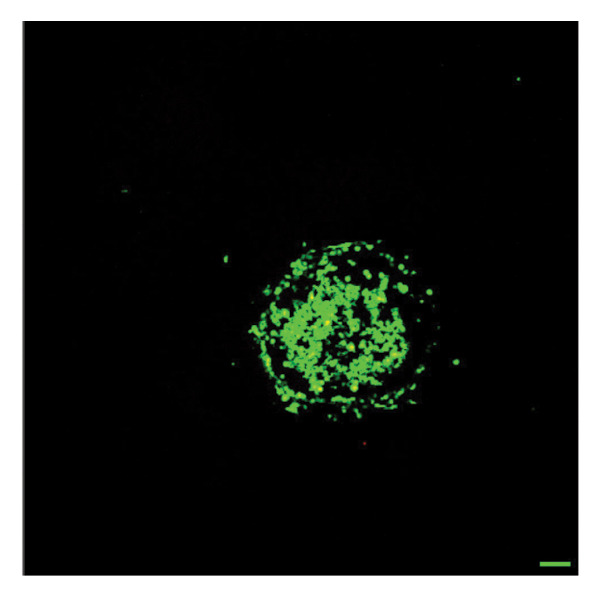
(b)

**Figure:**
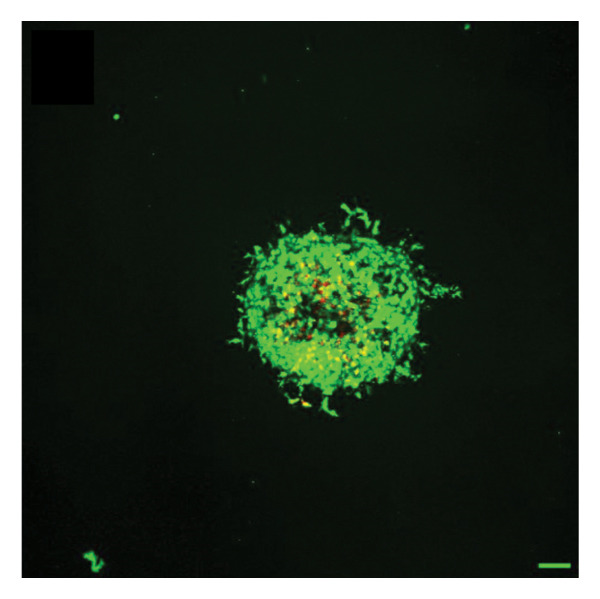
(c)

**Figure:**
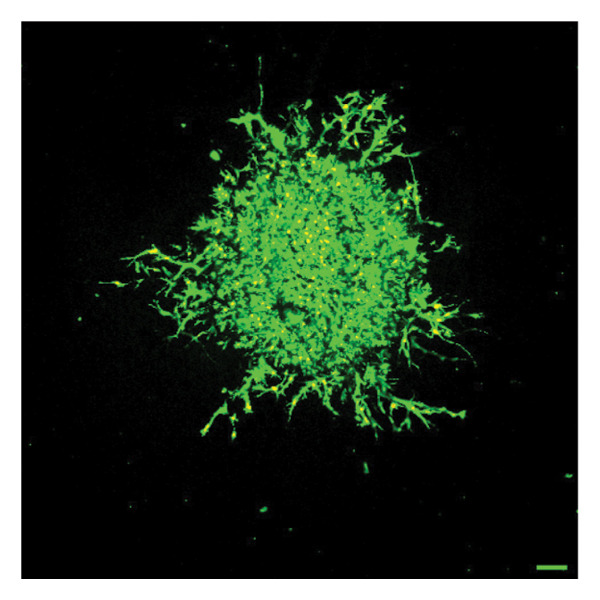
(d)

**Figure:**
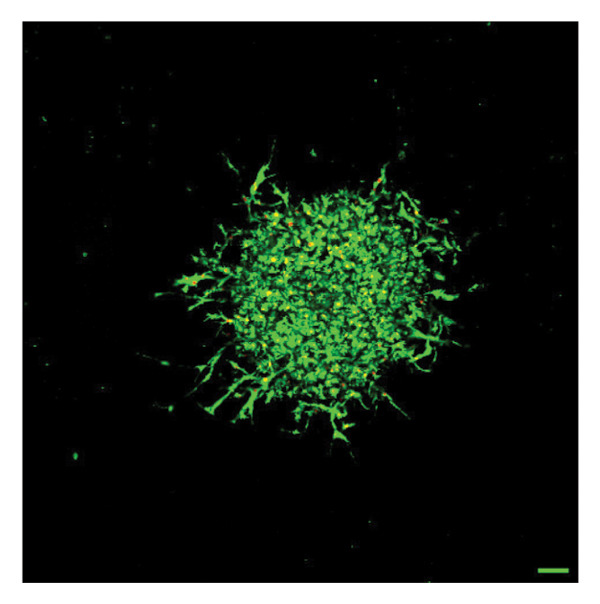
(e)

**Figure:**
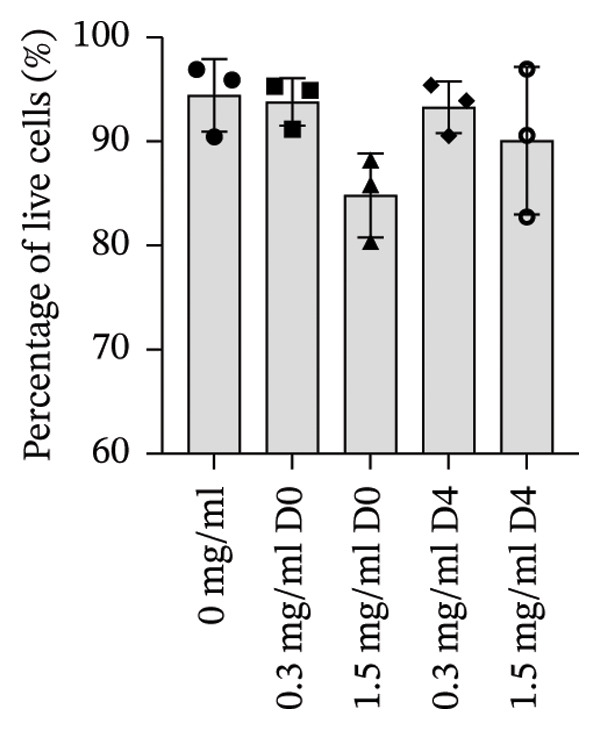
(f)

FIGURE 8(a) Representative phase contrast images of vascular sprouting of HUVEC/SMC co‐culture spheroids taken at D0, D4, D7, and D14 following encapsulation in scaffolds as a function of PFD dosing (0.3 and 1.5 mg/mL) and initial timing of PFD addition (D0 versus D4) in culture media. Vascular sprouts indicated by red arrows (scale bar = 100 μm). Quantification of percent increase in vascular sprouting area at D14 as a function of PFD dosage and initial timing of PFD addition at (b) D0 and (c) D4 (*n* = 3; *p* < 0.0065 (^∗^), *p* < 0.0001 (^∗∗^).

**Figure:**
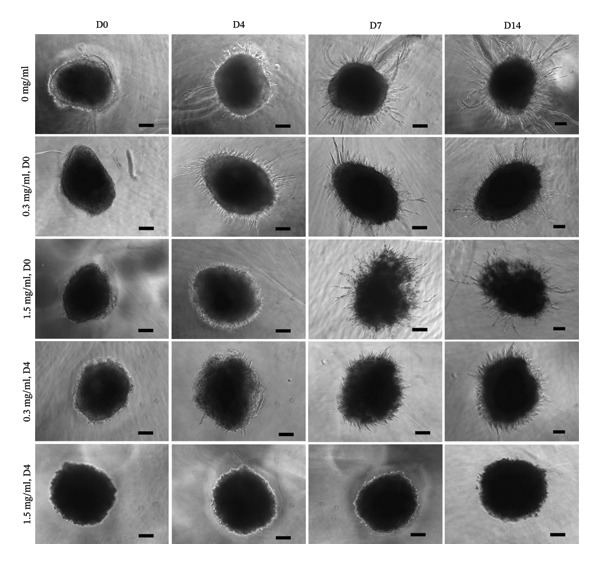
(a)

**Figure:**
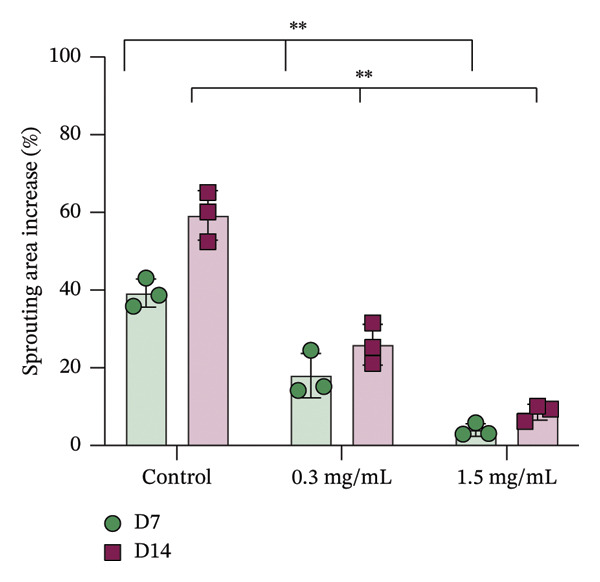
(b)

**Figure:**
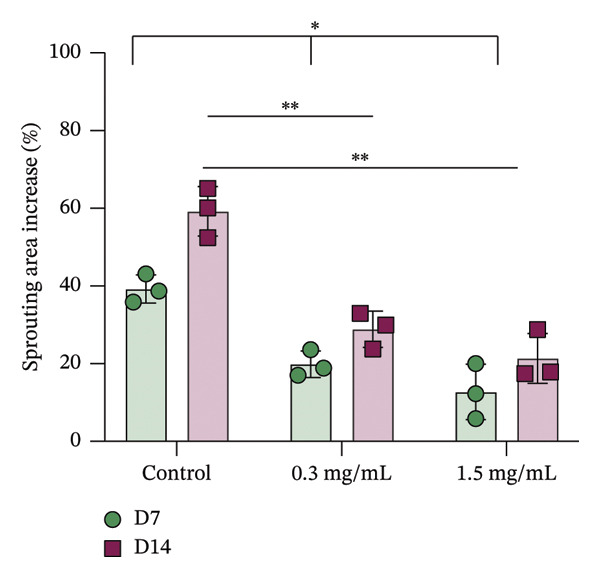
(c)

FIGURE 9Effect of PFD dose and timing of administration in culture media on vascular sprouting and viability at D14. Two‐dimensional projections of z‐stack confocal images of HUVEC/SMC 3D vascular sprouting under the following conditions: (a) 0 mg/mL PFD (control); PFD added on D0 at (b) 0.3 mg/mL and (c) 1.5 mg/mL; PFD added on D4 at (d) 0.3 mg/mL and (e) 1.5 mg/mL. (f) Quantification of the percentage of viable cells at D14 as a function of PFD dose and timing of administration. Viable and dead cells were visualized using calcein AM (green) and ethidium homodimer‐1 (EthD‐1, red), respectively (scale bar = 100 μm; *n* = 3; *p* < 0.05 (^∗^), *p* < 0.0001 (^∗∗^)).

**Figure:**
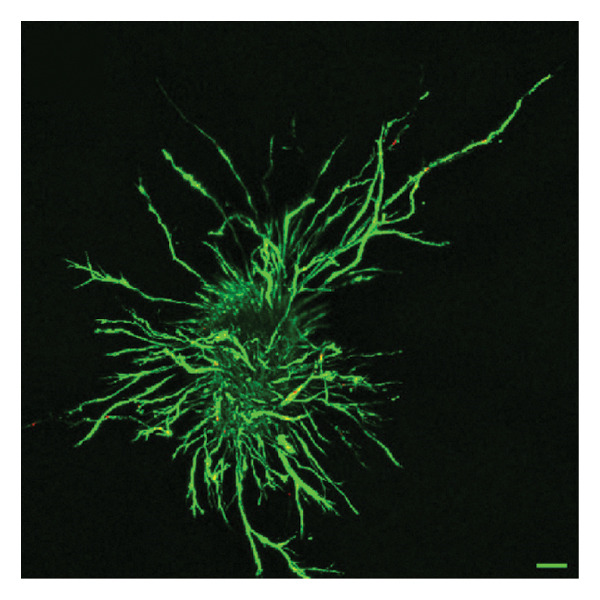
(a)

**Figure:**
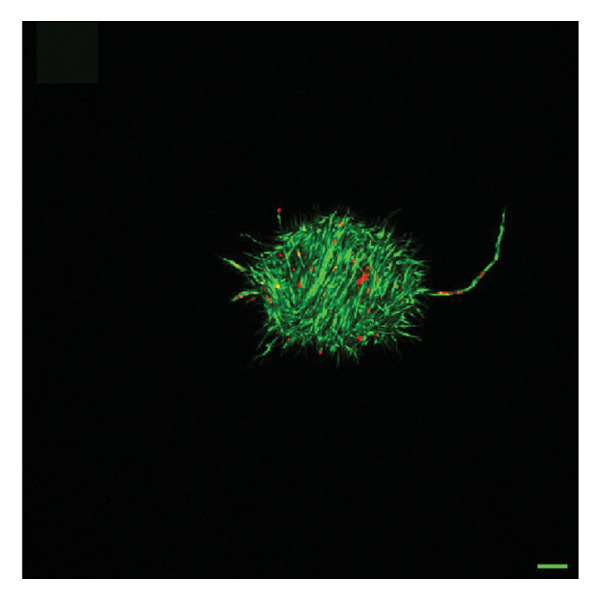
(b)

**Figure:**
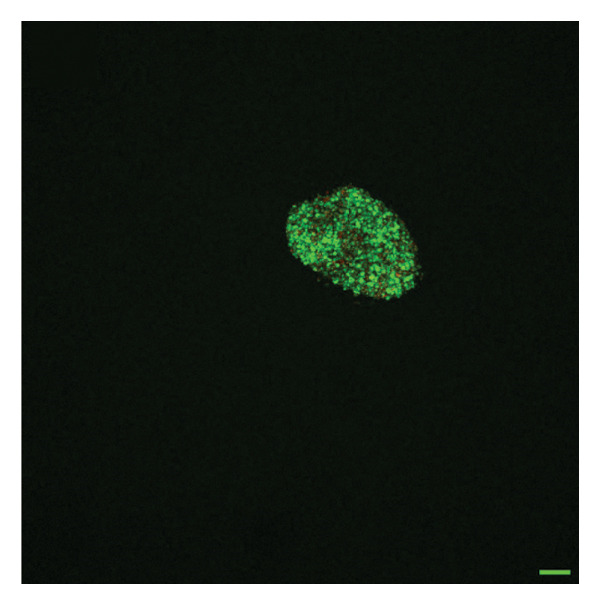
(c)

**Figure:**
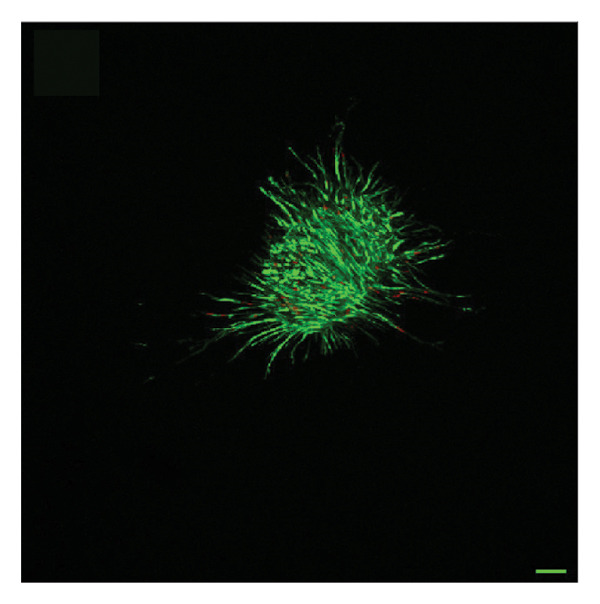
(d)

**Figure:**
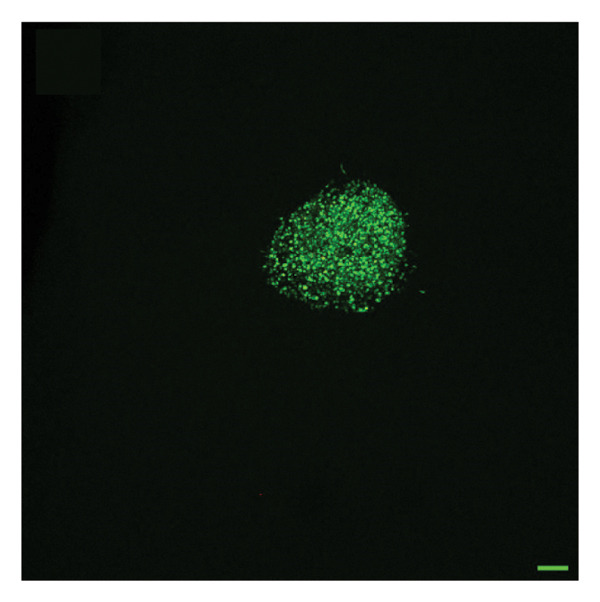
(e)

**Figure:**
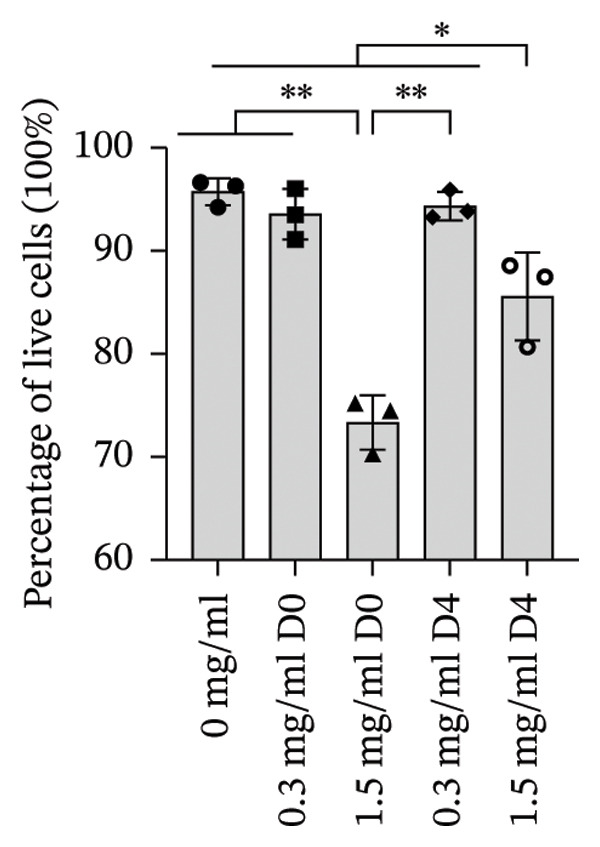
(f)

The effects of PFD dosage and timing of administration on fibroblast and vascular cell viability were quantified at D14 using confocal microscopy (Figures 7 and 9, respectively). Minimal impact on fibroblast viability was observed at the lower PFD dose, regardless of the timing of initial drug addition in culture. As shown in Figure 7(f), fibroblast spheroids treated with 0.3 mg/mL PFD exhibited viabilities of ∼93.8% and ∼93.3% when dosing began at D0 or D4, respectively, compared to ∼94.4% for the untreated control. At the higher PFD concentration of 1.5 mg/mL, the average fibroblast viability was reduced to ∼84.8% and ∼90.1% when PFD treatment began at D0 or D4, respectively. In HUVEC/SMC spheroid co‐cultures, the untreated control group exhibited a viability of ∼95.7%. Minimal changes in cell viability were observed with the addition of 0.3 mg/mL PFD, with viability reaching ∼93.6% and ∼94.4% when the drug was added to the culture media starting at D0 or D4, respectively. In contrast, the higher PFD dose (1.5 mg/mL) resulted in reduced viability, with averages of ∼73.3% and ∼85.6% when dosing began on D0 or D4, respectively, by D14 (Figure 9(f)).

## 4. Discussion

RA and OA are common chronic diseases that target the musculoskeletal system, frequently leading to significant joint tissue damage. Although the underlying disease mechanisms and epidemiology of RA and OA differ, the synovium has been identified as a major proinflammatory driver in both conditions, particularly in the early and progressive disease stages, through the processes of fibrotic and angiogenic remodeling [79–83]. Since current treatments for both diseases involving oral, systemic, and intra‐articular routes [84–95] are directed to control pain and inflammation, there is a critical need to identify novel therapeutics that mitigate fibrosis and angiogenesis to slow or prevent disease progression.

The use of FDA‐approved therapeutics already in clinical use presents a significant advantage over the development of new drugs, particularly for repurposing in refractory diseases such as RA and OA [22, 96]. Recent studies have focused on establishing in vitro models to investigate the biological mechanisms by which PFD targets synovial fibrosis and angiogenesis, with the goal of slowing OA [25] and RA [22, 61] progression. For example, in vitro antifibrotic and anti‐inflammatory effects of PFD have been reported in TGFb1‐stimulated FLS isolated from OA patients [25]. In 2D culture, PFD significantly inhibits FLS proliferation after 24 h at a concentration of 2.0 mg/mL compared to the untreated controls [25]. This effect was dose‐dependent across a concentration range of 0–2.0 mg/mL, with continued suppression observed at 48 and 72 h post‐treatment. A concentration of 1.0 mg/mL PFD inhibited FLS at 24 h and reduced the expression of fibrosis‐related gene COL1A1 and inflammatory cytokine IL6 at 72 h post‐treatment. Since a prime target of PFD is the inhibition of Smad2/3‐mediated TGFb1 signaling, it is likely that its mechanism of action includes a reversal of the myofibroblast activity to that of a nonpathogenic fibroblast phenotype [97].

Other studies have evaluated the role of PFD in suppressing synovial inflammation and angiogenesis in RA [22, 61]. In TNFα‐stimulated FLS, PFD significantly reduced mRNA and protein expressions of matrix metalloproteinases (MMP‐1, MMP‐2, MMP‐3, and MMP‐9), proinflammatory (IL1b, IL6, and IL8) markers, and the angiogenic cytokine VEGF [22]. PFD also exhibited antiangiogenic activity in vascular endothelial cells (EA.hy926), inhibiting Matrigel‐based surface tubulogenesis, 2D migration, and phosphorylation of JAK2/STAT3 and AKT pathways, key mediators of RA joint destruction [22]. These effects were dose‐dependent within the 0–2 mg/mL range which aligns with the PFD concentrations used in the 3D spheroid culture experiments presented herein.

Although previous studies have highlighted the importance of specific signaling pathways through which PFD attenuates synovial fibrosis and angiogenesis, most of these investigations have been limited to 2D culture assays and relatively short time frames (up to 72 h). To better approximate the inflamed synovium environment, where fibrosis and angiogenesis are already established, we evaluated the antifibrotic and antiangiogenic effects of PFD as a function of concentration and timing of initial administration (D0 vs. D4) using 3D spheroid culture models of fibrosis and neovascularization, with the goal of determining whether these processes are not only mitigated but also reversed after their onset.

Hydrogel matrices are often used to study drug diffusion. Our primary motivation for employing the spheroid‐laden hydrogel platform was not to regulate PFD diffusion, but to model the fibrotic and angiogenic 3D microenvironment characteristic of synovial inflammation and remodeling to screen the efficacy of the drug in mitigating fibrosis and angiogenesis. The cell adhesive, proteolytically degradable PEG hydrogels investigated in this study were designed to support robust fibroblast outgrowth [55] and vascular sprouting [10], behaviors that cannot be reproduced in 2D culture. We acknowledge that the mechanical properties and network mesh dimensions of crosslinked hydrogels, such as those used in the present study, also impact drug diffusivity. This effect, however, becomes negligible when the solute hydrodynamic diameter is significantly smaller compared to network mesh dimensions. In previous studies, we demonstrated that increases in the DSite‐PEGDA crosslinker concentration from 2.0 mM to 2.5 mM leads to a two‐fold increase in Young’s modulus from 2.11 ± 0.29 kPa to 4.01 ± 0.19 kPa [10]. This perturbation represents a physiologically relevant transition from healthy to early remodeled synovial tissue, consistent with the 0.5–5 kPa modulus range routinely employed in synovial mechanobiology studies in the early to pathologic range [58, 83]. Swelling ratio measurements and mesh size analyses (Supporting Figure 6) reveal that even after the scaffold elastic modulus is doubled, the hydrogel mesh dimensions remain orders of magnitude larger than the hydrodynamic diameter of PFD (0.93 nm). In the case of the PEG scaffolds used in this in vitro study (*E* = 2.11 ± 0.29 kPa), the hydrogel mesh dimensions *ξ*_*F* *R*_,​ *ξ*_*I* *C*_, *ξ*_*N* *C*_, are 51–73 ×, 103–165 ×, and 167–218 × larger than the PFD hydrodynamic diameter. When the modulus is doubled (*E* = 4.01 ± 0.19 kPa), these values remain 27–45 ×, 63–102 ×, and 101–147 × larger than the PFD hydrodynamic diameter (Supporting Figure 6). Across this stiffness range, PFD encounters no steric hindrance due to its small hydrodynamic size and is not retained within the hydrogel network, as the scaffold does not function as a drug depot (neither drug‐releasing nor drug‐absorbing). PFD is therefore supplied exogenously through the culture medium and replenished every other day to maintain continuous exposure to the embedded spheroids within the hydrogel, as its concentration would otherwise decrease due to diffusion and depletion within the hydrogel and the surrounding culture medium since the mesh dimensions of the hydrogel network far exceed the PFD hydrodynamic diameter. Maintaining defined exposure levels in vitro requires repeated replenishment and achieving a similar sustained dosage in vivo would require a sustained‐release delivery system. Thus, the dose–response insights derived from this 3D culture study will directly inform the rational design of controlled‐release formulations capable of maintaining PFD within the therapeutic concentration ranges identified in these assays.

PFD is a synthetic pyridone of relatively low molecular weight (185 Da) and hydrodynamic diameter (0.93 nm) compared to hydrogel mesh dimensions. Conservative estimates of the effective diffusion coefficient (*D*_*g*_/*D*_0_) of PFD obtained using the Flory–Rehner mesh dimensions and the Lustig–Peppas equation [73] for hydrogels of low and high modulus result in *D*_*g*_/*D*_0_ ≈ 0.959 and *D*_*g*_/*D*_0_ ≈ 0.939, respectively. When the effective diffusion coefficient is multiplied by the diffusion coefficient of PFD in water, *D*_0_≈6.98∗10^−6^ cm^2^/*s*, the diffusion coefficient values of PFD in gels of low and high modulus are *D*_*g*_ ≈ 6.70 × 10^−6^ cm^2^/*s* and *D*_*g*_ ≈ 6.55 × 10^−6^ cm^2^/*s*, respectively (Supporting Table 1). These values reflect mesh size estimates derived from the Flory–Rehner/Lustig–Peppas analysis. While fluorescence recovery after photobleaching (FRAP) provides a direct method for measuring diffusivity in polymer networks, it is more appropriate for future studies aimed at characterizing PFD transport in gels developed as sustained‐release depots. Our results indicate that the PFD gel diffusion coefficient is 4.1%–6.1% lower than that in pure water. Thus, the two‐fold increase in PEG elastic modulus results in only a ∼2.1% reduction in PFD diffusivity from 0.959 to 0.939. Such modest reductions occur because the size ratio of the PFD radius relative to the mesh dimension, *r*_*s*_/*ξ*_*F* *R*_, remains in the 0.009–0.014 range. These data demonstrate that within the physiologically relevant modulus range of PEG scaffolds (∼1–5 kPa) that overlaps values reported for synovial tissue, PFD diffusivity is essentially unaffected by matrix stiffness. In the case of OA and RA, intra‐articular injection of PFD would be prone to rapid clearance when administered via intra‐articular injection to an inflamed synovium, due to enhanced and vascular [98] and lymphatic drainage [99]. Consequently, sustained or repeated delivery would likely be required to maintain therapeutic efficacy and ensure adequate drug levels in the synovial tissue if PFD were to be repurposed as a targeted treatment for joint inflammation. Our ongoing and future studies aim to address this by developing an injectable intra‐articular drug delivery system for targeted and sustained release of PFD.

## 5. Conclusions

Our 3D culture findings demonstrate that PFD significantly attenuates fibroblast spheroid outgrowth compared to the untreated control group, whether initially administered at D0 or D4 (Figure 6). The antifibrotic effects were not dose‐dependent when PFD was introduced at D0, whereas dose‐dependence was observed with PFD initial treatment at D4. Across all conditions investigated, fibroblast spheroid viability was maintained above 80% (Figure 7(f)). The dose‐dependence observed with PFD administration at D4 may be linked to the higher percentage of cell viability over the concentration range investigated, consistent with previous findings showing that fibroblasts maintain higher viability across a broader range of PFD concentration (0–3.7 mg/mL) [22]. PFD was also found to attenuate vascular sprouting in HUVEC/SMC spheroid co‐cultures, regardless of whether it was administered immediately after spheroid encapsulation (D0) or after 4 days (D4), compared to the untreated control. Attenuation in vascular sprouting was found to be dose‐dependent when PFD was introduced at D0, but not at D4 (Figure 8). This outcome may be due to the observed reduction in vascular spheroid viability at the higher PFD concentration of 1.5 mg/mL, which aligns with previous studies reporting reduced drug viability of endothelial cells, although this concentration was not found to be cytotoxic [22, 61]. Regardless of the timing of PFD administration or concentration, the viability of HUVEC/SMC spheroids was maintained over 70% (Figure 9(f)). Notably, the lower concentration of 0.3 mg/mL PFD was sufficient to significantly attenuate both fibroblast outgrowth and vascular sprouting. In addition, our results indicate that the addition of PFD throughout 3D spheroid cultures up to D14 attenuates both fibroblast invasion and neovascularization regardless of timing of administration and reverses the onset of fibrosis and neovascularization at the higher PFD dose of 1.5 mg/mL.

Various spheroid culture platforms have been developed as in vitro models of inflammatory arthritides to elucidate the complex 3D interactions between cells and proinflammatory mediators present in the inflamed synovium. These in vitro platforms have also been utilized to screen therapeutics used in clinical management of pain and inflammation [14]. Triculture spheroids consisting of RA‐derived FLS, HUVECs, and monocyte‐derived macrophages embedded in collagen‐based scaffolds have demonstrated robust cell–cell interactions and spheroid outgrowth in response to VEGF stimulation [100]. Although such models effectively replicate the multicellular and proinflammatory microenvironment of the inflamed synovium, they pose challenges for mechanistic screening of pathways through which novel therapeutics mitigate fibrosis and angiogenesis. In future studies, we aim to further explore the pathways through which sustained delivery of PFD from polymer nanoparticles mitigates synovial fibroblast activation, invasion, and proliferation, as well as neovascularization in 3D culture and animal models of arthritis.

## Funding

Research resulting from this publication was supported by the National Institutes of Arthritis and Musculoskeletal and Skin Diseases of the NIH under award number R21AR074072 (Georgia Papavasiliou, PI) and the Pritzker Institute of Biomedical Science and Engineering Graduate Fellowship Program.

## Conflicts of Interest

The authors declare no conflicts of interest.

## Supporting Information

We have included Supporting Information for this paper, which provides additional data and methods complementing the main text. Supporting Figure 1 presents the time sweep evolution of storage (*G* ^′^) and loss (*G* ^″^) moduli for hydrogels formed with 2 mM and 2.5 mM DSite‐PEGDA crosslinker measured at a strain amplitude of 0.05% and *ω* = 10 rad/s. Supporting Figure 2 presents the standard curve of PFD absorbance versus concentration used to quantify kinetics of PFD scaffold uptake. Supporting Figure 3 presents the methodology used to quantify 3D spheroid invasion and vascular sprouting from automated phase contrast microscopy image processing, including pre‐processing, segmentation, and invasion zone identification. Supporting Figures 4 and 5 display 3D renderings of z‐stack confocal images depicting fibroblast outgrowth and vascular sprouting within the scaffolds, respectively, at D14 for untreated and PFD‐treated conditions (PFD added at D0 or D4). Supporting Figure 6 shows the effects of DSite‐PEGDA crosslinker concentration (2 mM and 2.5 mM) on hydrogel network mesh dimensions, demonstrating increased elastic modulus and reduced mesh size at higher crosslinking concentration. Supporting Table 1 provides the estimated PFD diffusivity values as a function of hydrogel modulus and mesh dimensions.

## Supporting information


**Supporting Information** Additional supporting information can be found online in the Supporting Information section.

## Data Availability

The data that support the findings of this study are available from the corresponding author upon reasonable request.
