# Supplementary material for: Pirfenidone Attenuates Fibrosis and Neovascularization in 3D Spheroid‐Laden Hydrogel Culture
Source: J Tissue Eng Regen Med. 2026 Apr 15;2026:5557686. doi: 10.1155/term/5557686 (PMC13080344; doi:10.1155/term/5557686)
Supplement: Supplementary file 1 — Supporting Information Additional supporting information can be found online in the Supporting Information section. [file TERM-2026-5557686-s001.zip › Supplementary Section.pdf]

Supplementary Section

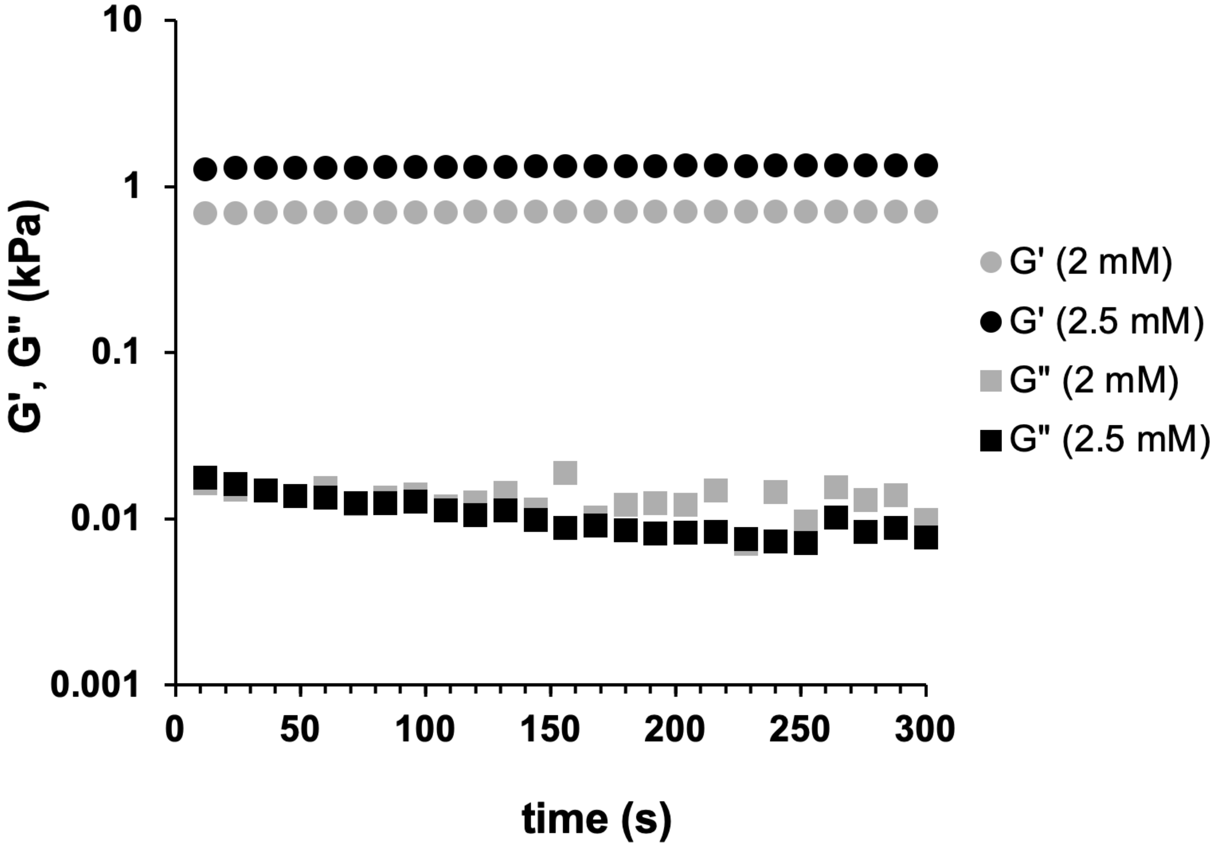

Supplementary Fig. 1. Time-sweep of storage (G′) and loss (G″) moduli for hydrogels formed with 2 mM and 2.5 mM DSite PEGDA crosslinker measured at a strain amplitude of 0.05% and ω = 10 rad/s.

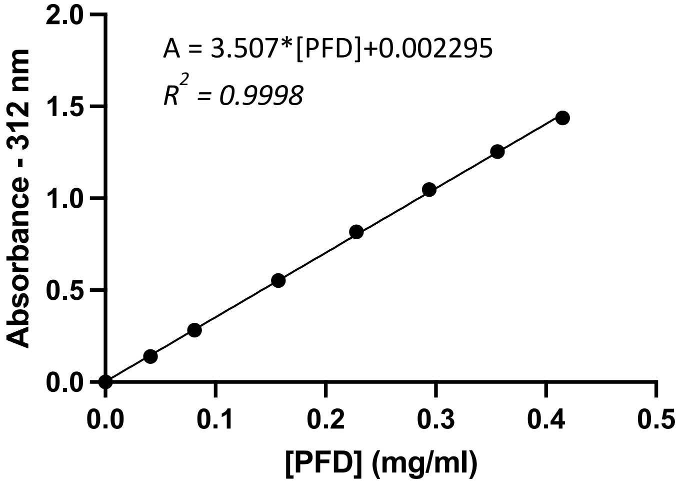

Supplementary Fig. 2. Standard curve of absorbance versus PFD concentration used to quantify kinetics of PFD scaffold uptake.

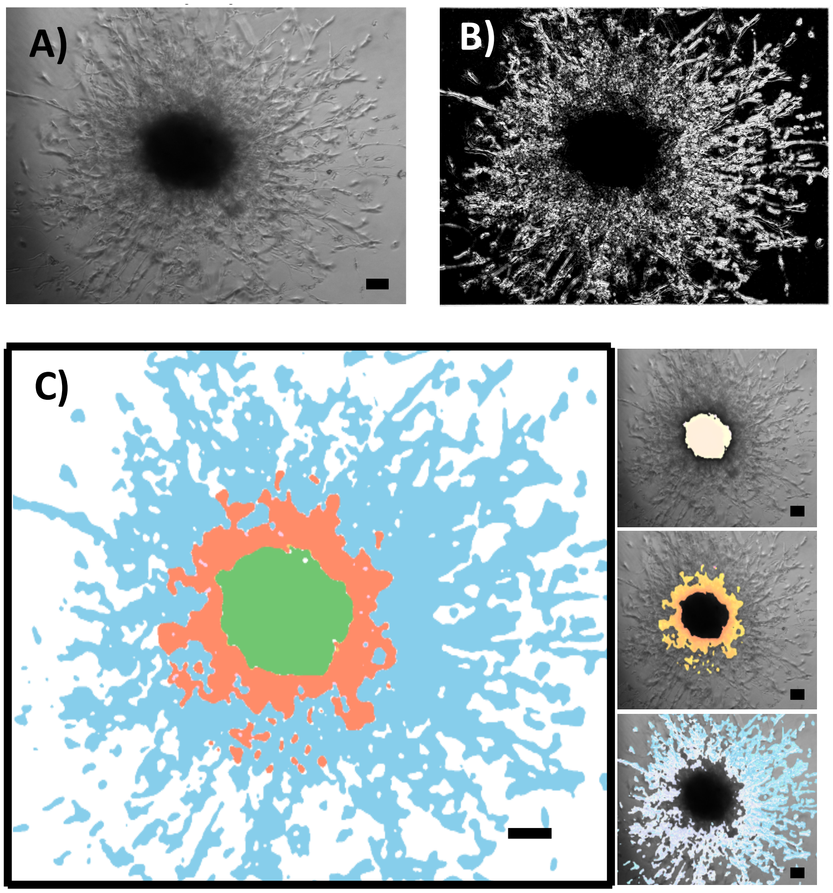

Supplementary Fig. 3. Methodology used to quantify dynamics of 3D spheroid invasion and vascular sprouting using automated phase contrast image processing (representative images shown for HUVEC/SMC spheroid vascular sprouting): (A) Original phase contrast image displaying the invasion of vascular sprouts within hydrogel scaffolds; (B) Enhanced image post-deconvolution, noise reduction and diffusion; (C) Segmentation analysis distinguishing spheroid core (green) and cell invasion zones (orange and blue). (scalebar =100mm)

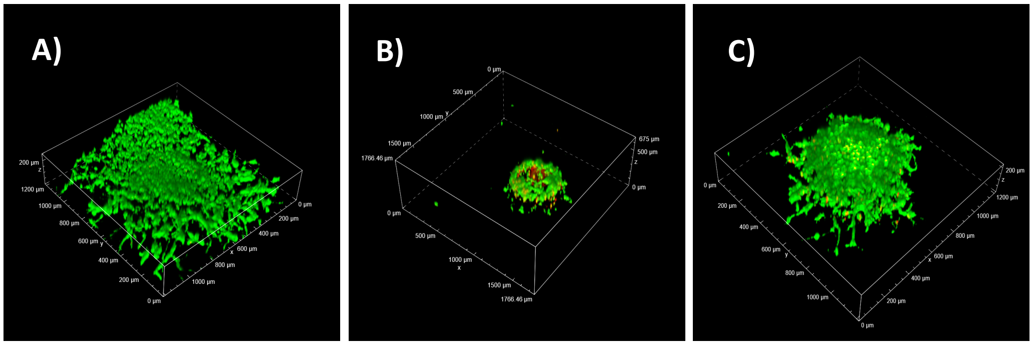

Supplementary Fig. 4. 3D renderings of z-stack confocal images of 3D fibroblast outgrowth at D14 of (A) 0 mg/mL PFD; 1.5 mg/mL PFD added on (B) D0 and (C) D4. Calcein-AM (green) and Eth1 (red) used to image and quantify viable and dead cells, respectively.

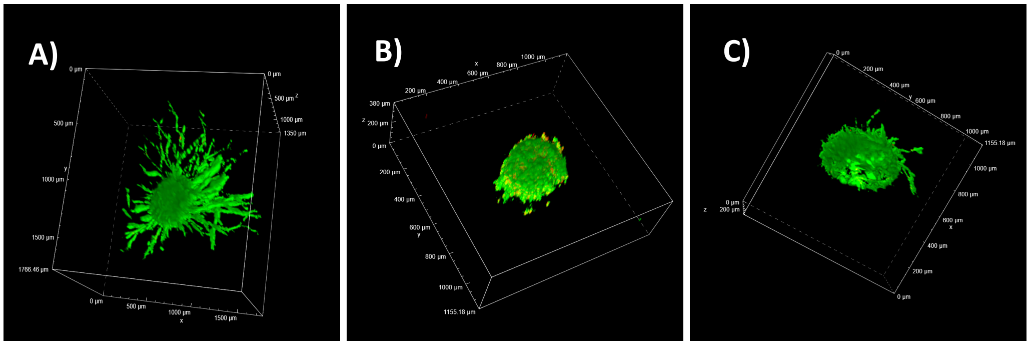

Supplementary Fig. 5. 3D renderings of z-stack confocal images of HUVEC/SMC 3D vascular sprouting at D14 with (A) 0 mg/mL PFD; 1.5 mg/mL PFD added on (B) D0 and (C) D4. Calcein-AM (green) and Eth1 (red) used to image and quantify viable and dead cells, respectively.

To determine the effect of scaffold mechanical properties on PFD hydrogel diffusivity and the resultant network mesh dimensions, PEG hydrogels were synthesized by visible light free-radical photopolymerization using varying concentrations (2.0 mM and 2.5 mM) of the DSite PEGDA crosslinker as previously described.[11] This resulted in scaffolds with Young's moduli of 2.11 ± 0.29 kPa and 4.01 ± 0.19 kPa, for the 2.0mM and 2.5 mM precursor formulations, respectively.

Gravimetric measurements in mass and volumetric swelling ratios for hydrogels synthesized with varying crosslinker concentration were then used to obtain the network mesh dimensions (ξ_{FR}, ξ_{IC}, ξ_{NC}) as described in Materials and Methods (Supplementary Fig. 6). The diffusion coefficients of PFD were estimated from the resultant Flory-Rehner mesh sizes (ξ_{FR}) using the Lustig-Peppas equation (Supplementary Table 1). [74]

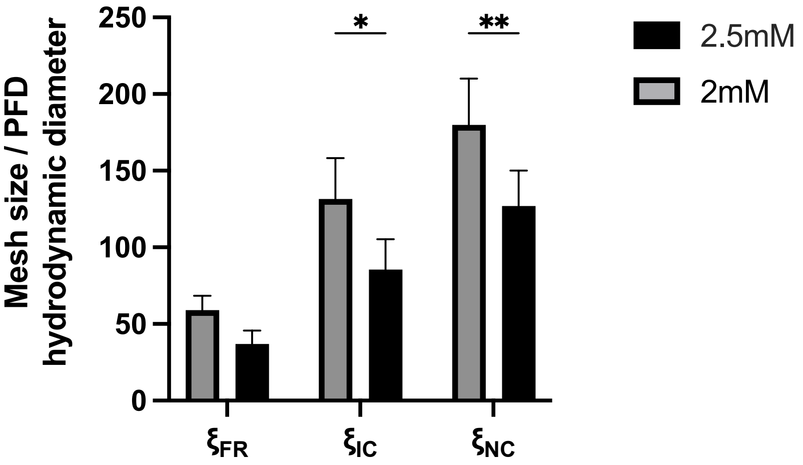

Supplementary Fig. 6. Effect of DSite-PEGDA crosslinker concentration (2mM and 2.5 mM) on hydrogel network mesh dimensions. Increasing crosslinker concentration results in increases hydrogel elastic modulus and decreases in mesh dimensions (* = $p \leq 0.05$, ** = $p \leq 0.01$, n=4).

Supplementary Table 1. Effect of PEG hydrogel modulus and mesh dimensions on the PFD diffusivity.

**Formulation**	**E (kPa)**	ξ_{FR}**(nm)**	ξ_{IC}**(nm)**	ξ_{NC}**(nm)**	D_g/D_0	D_g **(cm^2/s)**
2.0 mM DSite-PEGDA	2.11 ± 0.29	53.6 ± 8.3	118.0 ± 23.6	162.0 ± 26.3	0.96	6.70x10^{-6}
2.5 mM DSite-PEGDA	4.01 ± 0.19	33.2 ± 6.7	76.4 ± 15.5	114.1 ± 20.9	0.94	6.55x10^{-6}
