# Supplementary material for: Pirfenidone Attenuates Fibrosis and Neovascularization in 3D Spheroid‐Laden Hydrogel Culture
Source: J Tissue Eng Regen Med. 2026 Apr 15;2026:5557686. doi: 10.1155/term/5557686 (PMC13080344; doi:10.1155/term/5557686)
Supplement: Supplementary file 1 — Supporting Information Additional supporting information can be found online in the Supporting Information section. [file TERM-2026-5557686-s001.zip › Supplementary.pdf]

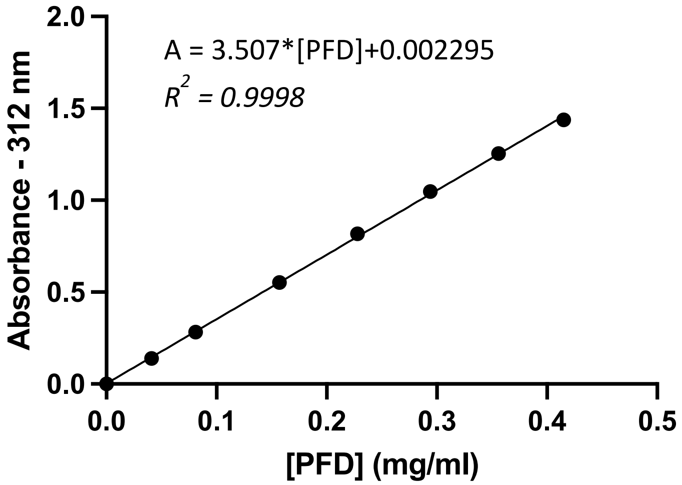

Supplementary Fig. 1. Standard curve of absorbance versus PFD concentration used to quantify kinetics of PFD scaffold uptake shown in Figure 5.

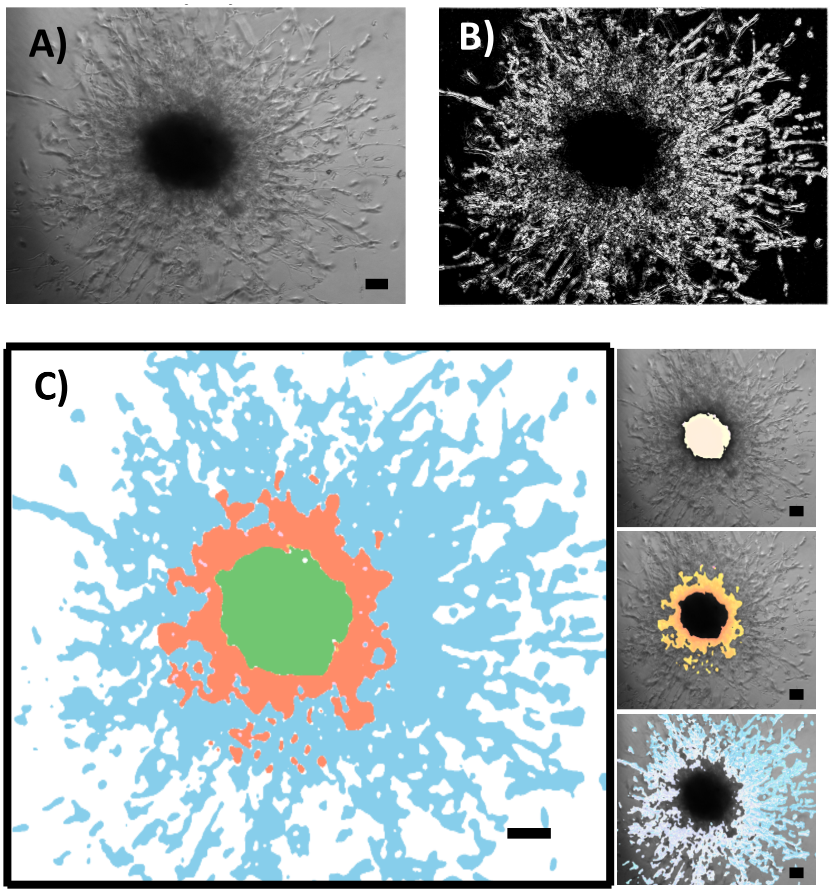

Supplementary Fig. 2. Methodology used to quantify dynamics of 3D spheroid invasion and vascular sprouting using automated phase contrast image processing (representative images shown for HUVEC/SMC spheroid vascular sprouting): (A) Original phase contrast image displaying the invasion of vascular sprouts within hydrogel scaffolds; (B) Enhanced image post-deconvolution, noise reduction and diffusion; (C) Segmentation analysis distinguishing spheroid core (green) and cell invasion zones (orange and blue). (scalebar =100mm)

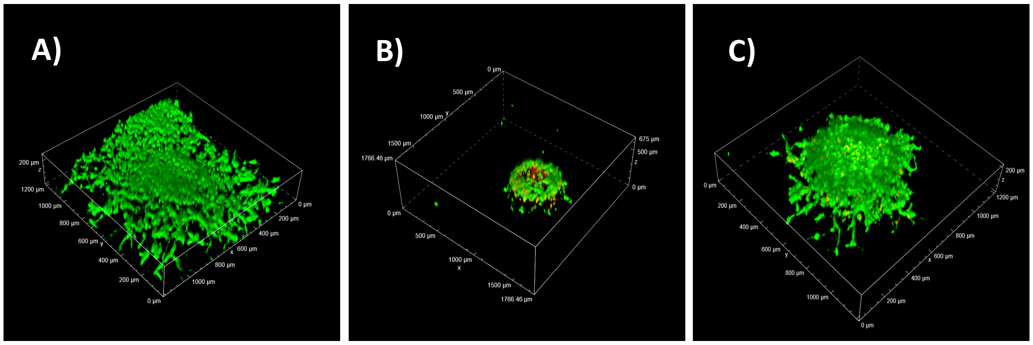

Supplementary Fig. 3. 3D renderings of z-stack confocal images of 3D fibroblast outgrowth at D14 of (A) 0 mg/ml PFD; 1.5 mg/ml PFD added on (B) D0 and (C) D4. Calcein-AM (green) and Eth1 (red) used to image and quantify viable and dead cells, respectively.

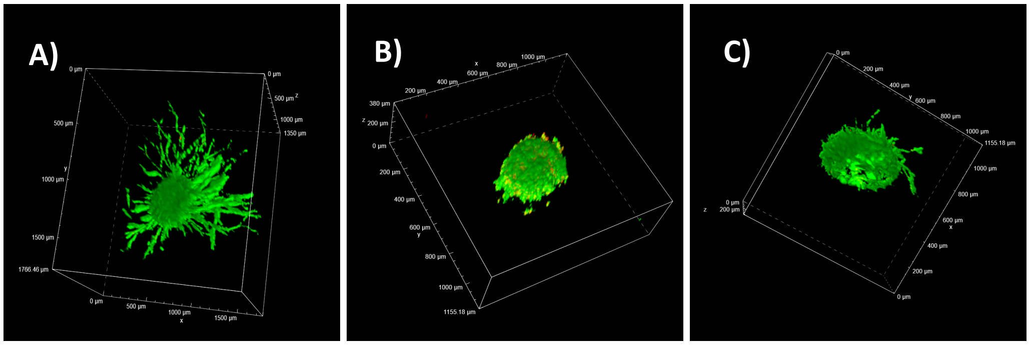

Supplementary Fig. 4. 3D renderings of z-stack confocal images of HUVEC/SMC 3D vascular sprouting at D14 with (A) 0 mg/ml PFD; 1.5 mg/ml PFD added on (B) D0 and (C) D4. Calcein-AM (green) and Eth1 (red) used to image and quantify viable and dead cells, respectively.
